# As a Commentary on a Certain Class of “Regular” Advertising

**Published:** 1881-02

**Authors:** 


As a commentary on a certain class of "regular" advertising, we publish the following from the Boston *Medical and Surgical Journal:* Dr. T. Gaillard Thomas's beautiful new private hospital for the treatment of diseases peculiar to women, at the corner of Lexingten Avenue and Fifty-Second street, has now been opened, and any patient admitted there will be sure to have every possible provision made for her comfort as well as to receive such treatment as the highest skill and science can suggest. The resident surgeon is Dr. James B. Hunter, of the attending staff of the State Woman's Hospital, and his own private office is located in the building.

